# The marine Omega3 wound matrix for treatment of complicated wounds: A multicenter experience report

**DOI:** 10.1007/s00772-018-0428-2

**Published:** 2018-08-01

**Authors:** B. Dorweiler, T. T. Trinh, F. Dünschede, C. F. Vahl, E. S. Debus, M. Storck, H. Diener

**Affiliations:** 1 Vascular Surgical Unit, Department of Cardiac, Thoracic, and Vascular Surgery, Mainz University Hospital, Langenbeckstraße 1, 55131 Mainz, Germany; 2 Department and Outpatient Clinic for Vascular Medicine, University Heart Center Hamburg, Hamburg-Eppendorf University Hospital, Hamburg, Germany; 3 Department of Vascular and Thoracic Surgery, Karlsruhe Hospital, Karlsruhe, Germany

**Keywords:** Diabetic ulcer, Arterial occlusive disease, Wound healing, Wound matrix, Amputation, Diabetisches Ulkus, Arterielle Verschlusserkrankung, Wundheilung, Wundmatrix, Amputation

## Abstract

**Introduction:**

The Kerecis™ Omega3 Wound matrix is a decellularized skin matrix derived from fish skin and represents an innovative concept to achieve wound healing. The aim of this study was to report the cumulative experience of three centers for vascular surgery regarding use of the Omega3 Wound matrix in selected patients with complicated wounds.

**Material and methods:**

In this study 23 patients with 25 vascular and/or diabetes mellitus-associated complicated wounds and partially exposed bony segments were treated with the Omega3 Wound matrix in three vascular centers. In several patients, conventional wound treatment with vacuum therapy had previously been carried out sometimes over several weeks without durable success. Following initial debridement in the operating room, the matrix was applied and covered with a silicone mesh. In the further course, wound treatment was conducted on an outpatient setting if possible.

**Results:**

In total 25 wounds were treated with localization at the level of the thigh (*n* = 2), the distal calf (*n* = 7), the forefoot (*n* = 14) and the hand (*n* = 2). The time to heal varied between 9 and 41 weeks and between 3 and 26 wound matrices were applied per wound. Interestingly, a reduction of analgesics intake was noted when the treatment with the Omega3 Wound matrix was initiated.

**Conclusion:**

The novel Omega3 Wound matrix in this study represented an effective treatment option in 25 complicated wounds. Further studies are necessary to evaluate the impact of the wound matrix on stimulation of granulation tissue and re-epithelialization as well as the potential antinociceptive and analgetic effects.

## Zusammenfassung

**Einleitung:**

Die Omega-3-Wundmatrix (Kerecis®) stellt eine innovative Wundauflage dar, die aus der Haut des Kabeljaus durch entsprechende Verfahren wie Dezellularisierung gewonnen wird und seit Kurzem in Deutschland verfügbar ist. Die Intention der vorliegenden Studie ist die Zusammenstellung der Behandlungserfahrungen aus 3 gefäßchirurgischen Zentren mit dem Einsatz der Omega-3-Wundmatrix bei selektierten Wundpatienten.

**Material und Methoden:**

In einem multizentrischen Erfahrungsbericht haben wir die Ergebnisse von insgesamt 23 Patienten mit 25 vaskulären und diabetes- bzw. vaskulitisassoziierten Problemwunden zusammengestellt. Bei mehreren Patienten war zuvor eine konventionelle Wundbehandlung mit Vakuumtherapie teils über mehrere Wochen ohne durchgreifenden Erfolg durchgeführt worden. Die Erstapplikation der Omega-3-Wundmatrix erfolgte im OP unter stationären Bedingungen, danach wurden die Patienten nach Möglichkeit einmal wöchentlich ambulant versorgt.

**Ergebnisse:**

Insgesamt behandelten wir 25 Problemwunden sowohl nach Amputationen als auch bei primärer Wunde (Oberschenkel *n* = 2, Unterschenkel *n* = 7, Fuß *n* = 14, Hand *n* = 2). Die Abheilungszeiten variierten von 9 bis 41 Wochen, die Anzahl der pro Wunde verwendeten Omega-3-Wundmatrizes von 3 bis 26. Zusätzlich war im Rahmen der Behandlung mit Omega-3-Wundmatrizes eine Verminderung der lokalen Wundschmerzen zu verzeichnen.

**Schlussfolgerung:**

Die Behandlung mit der innovativen marinen Omega-3-fettsäurehaltigen Wundmatrix stellt in dieser Untersuchung einen wirksamen Ansatz in der Behandlung von 25 Problemwunden dar. Weitere Untersuchungen sind notwendig, um die Granulations- und Reepithelialisierungsfunktion sowie die zusätzlich vorhandene analgetische bzw. antinozizeptive Wirkung dieses Präparates genauer zu evaluieren.

## Introduction

According to the latest estimates between 440,000 and 1.8 million patients currently suffer from chronic wounds in Germany. Wounds of this kind are of considerable sociodemographic relevance, not least due to the impaired quality of life of patients and the costs they generate. A further problem not to be underestimated is the often long healing process or in some cases, the failure to heal despite treatment of the underlying disease and optimization of local therapy [1].

With a prevalence of 2–10%, diabetic foot syndrome (DFS) plays an important role in chronic wounds and, particularly in this context, the risk of limb loss due to amputation is at its highest (up to 60%; [2]). At present, only the efficacy of local wound debridement and ensuring a moist wound environment through appropriate wound dressings have been verified in the treatment of DFS (as in general in the treatment of chronic wounds); however, in terms of the various debridement techniques available (surgical, bio-surgical, enzymatic), there are no data on the superiority of any one of these procedures over the others [3]. Regarding vacuum therapy, it should be noted that there is no reliable (level I) evidence to support its use in the treatment of chronic wounds [4, 5].

Acellular dermal matrix preparations have been investigated as an alternative concept and have shown potential in individual studies as a promising adjunct to promote faster wound healing [6–9]. Extracellular matrix (ECM) preparations represent the third generation of wound dressings, with the vast majority of available matrices being of porcine and bovine origin (intestinal submucosa) or are produced from amniotic tissue. They serve as a matrix for cell proliferation and cell migration via their cytokines and growth factors, which promote cell proliferation and angiogenesis. At the same time, both inflammation and matrix metalloproteinases (MMP) are regulated and ECM peptides show reduced bacterial growth in vitro [10–12].

The porous microstructure enables the ingrowth of dermal cells and capillaries

The Kerecis® Omega3 wound matrix (Kerecis, Isafjordur, Iceland) is also an acellular dermal matrix; however, it is of marine origin and is derived from the skin of Atlantic cod (*Gadus morhua*). Due to the particularly gentle process of decellularization and preservation, the protein and matrix structure, which incidentally is extremely similar to the structure of human skin, remains intact and its porous microstructure enables the ingrowth of dermal cells and capillaries.

**Fig. 1:**
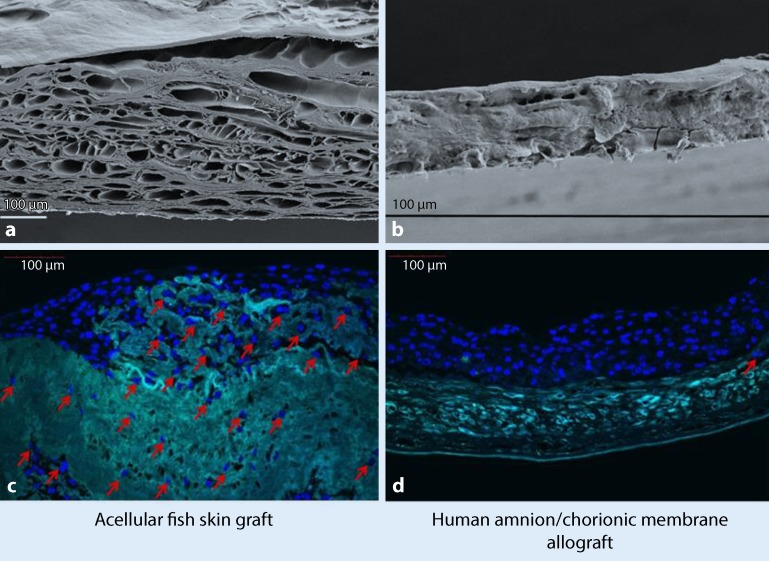
Ultrastructure of the matrix. **a,** **b** Acellular dermal matrix derived from fish skin (**a**) and decellularized amnion matrix (**b**) compared under a scanning electron microscope (SEM). **c,** **d** Stem cells were cultured for 12 days in an acellular dermal matrix derived from fish skin (**c**) and amnion (**d**). The stem cells stained *blue* (marked with *arrows*) have migrated into the fish skin matrix, while the stem cells on the amniotic matrix have settled on the surface of the matrix. (Used with kind permission from Kerecis®. This content is not part of the Open Access Licence)

Furthermore, the special preservation process used, which, in contrast to the decellularization processes of porcine and bovine matrices requires comparatively little denaturation, maintains the extremely high long-chain omega-3 fatty acid content [13, 14]. In the experimental setting, this results in an immunomodulatory effect by inhibiting macrophage secretion of the proinflammatory cytokine interleukin 1-beta. Bacterial activity against gram-positive and gram-negative bacteria, as well as antiviral activity against HIV and Herpes simplex viruses, has been demonstrated. In vitro studies showed increased stem cell migration and proliferation of decellularized fish skin in microscopically larger pores within the matrix compared with porcine matrices or amnion-derived matrices (Fig. 1). In addition, increased angiogenesis was demonstrated in the chick chorioallantoic membrane (CAM) assay in vivo. Early clinical applications in the treatment of chronic wounds point to improved wound healing compared with porcine ECM [13, 15]. The Omega3 wound matrix is available as a vacuum-dried preparation in a variety of sizes and is FDA-approved and CE-certified for the treatment of chronic wounds.

In this article the authors present their results with the application of the Omega3 wound matrix in the form of a multicenter experience report, whereby the results from the three centers are presented separately due to differences in patient selection and the respective treatment endpoints.

## Experience report from the University Hospital Mainz

### Methods

A total of eight patients were treated between November 2014 and April 2017 in this extension of the authors’ initial pilot series [16]; the inclusion criterion was a complicated wound on the lower limb following previous amputation with exposed bone and a risk of reamputation (proximalization). The average age of patients was 71 ± 8 years (Table 1) and all patients had at least two risk factors for cardiovascular disease.

**Table 1:** Patient characteristics and outcomes

Patient (M/F, age, years)	Wound size (cm^2^)	Wound location	History	Treatment duration (weeks)	Residual wound size (cm^2^)	Number of patches
Mainz						
F, 76	6/28	Proximal thigh	DFS, status following PTA/pelvic stent, popliteo-pedal bypass, below-knee amputation, above-knee amputation	16/26	0	26
M, 67	11	Metatarsus	DFS, status following popliteopedal bypass, TMA, secondary amputation metatarsus	30	0	18
M, 74	17	1st Ray	DFS, status following D1 amputation, secondary amputation MT1	41	0	20
M, 76	6/8	Forefoot	DFS, status following popliteopedal bypass, TMA	13/19	0	10
M, 80	29	Forefoot	DFS, PTA/pelvic stent, femorocrural bypass, TMA	33	0	22
M, 58	63	Forefoot	DFS, TMA	27	0	16
M, 62	35	Metatarsus	DFS, popliteopedal bypass, TMA, secondary amputation metatarsus	12	0	14
M, 72	24	Forefoot	DFS, popliteocrural bypass, amputation D1	9	0	7
Hamburg						
M, 53	11	Lower leg	PAOD, status following above-knee amputation	10	0	9
M, 53	6	D1 distal phalanx	PAOD, status following popliteal PTA	47	2	7
F, 78	12	Forefoot	DFS, status following popliteal PTA and forefoot amputation	23	0	3
M, 50	30	Lower leg	DFS, status following PTA/pelvic stent	12	0	4
M, 72	58	Distal lower leg	PAOD, PTA/pelvic stent	7 (patient deceased)	47	7
F, 72	47	Forefoot	PAOD, unsuccessful revascularization	73	15	17
M, 66	3	Forefoot	DFS, status following PTA of the posterior tibial artery and amputation 1st ray	16	0	4
F, 69	5	Lower leg	Vasculitis, status following above-knee amputation	4	2	3
F, 80	40	Distal lower leg	DFS	4	25	4
M, 80	16	Forefoot	DFS, status following pedal bypass	3	11	2
Karlsruhe						
F, 101	–	Forefoot	DFS	3	Partial healing	2
F, 80	–	Lower leg	PAOD, CVI, pelvic PTA, femoropopliteal bypass	16	0	2
M, 67	–	Lower leg	CVI	24	0	4
M, 62	–	Metacarpus	Ischemic necrosis (sepsis-related)	24	0	1
M, 74	–	Metacarpus	Ischemic necrosis (dialysis shunt-associated steal syndrome)	12	Partial healing	1

*CVI* chronic venous insufficiency, *DFS* diabetic foot syndrome, *MT1* metatarsal bone I, *PAOD* peripheral arterial occlusive disease, *TMA* transmetatarsal amputation, *PTA* percutaneous transluminal angioplasty

Sufficient peripheral perfusion was initially achieved surgically in six of the eight patients by placing a popliteopedal (*n* = 5) or femorocrural bypass (*n* = 1); however, bypass occlusion occurred in one patient that required firstly lower and then upper limb amputation following unsuccessful thrombectomy. Of the patients two had primarily adequate peripheral perfusion. Thus, all patients exhibited adequate arterial perfusion in the wound area at the start of treatment (ankle-brachial index ABI > 0.5 and/or transcutaneous partial pressure of oxygen [tcpO_2_] > 40 mm Hg). Reamputation and proximalization of the amputation level was performed in 6 (Chopart amputation *n* = 2, proximal transmetatarsal amputation *n* = 3, transmetatarsal amputation *n* = 1) of 8 patients due to impaired wound healing in the wound area following initial amputation: one patient [1] had already undergone secondary resection twice (distal bone, proximal bone). Therefore, all seven patients with impaired wound healing and partially exposed bony segments were at risk of conversion to major amputation (*n* = 6) or hip disarticulation.

Initial wound treatment comprised radical surgical debridement, with swabs taken intraoperatively under aseptic conditions. The Omega3 wound matrix was then cut according to wound size, soaked in physiological saline solution for 1 min, and then applied to the wound and covered using a silicone foam dressing (Mepilex® Border, Mölnlycke Health Care, Göteborg, Sweden). In the further course, wound treatment was continued in the outpatient setting with one visit per week. At each visit, the wound was cleaned or debrided, wound size was photodocumented, and any defects/zones of patch absorption filled with new Omega3 wound matrix.

The statistical analysis of patient data and the quantification of wound surface area were carried out using MS EXCEL (Microsoft Corp., Redmond, WA, USA) and Fiji/Image J open source software (imagej.nih.gov/ij/), respectively.

### Results

**Fig. 2:**
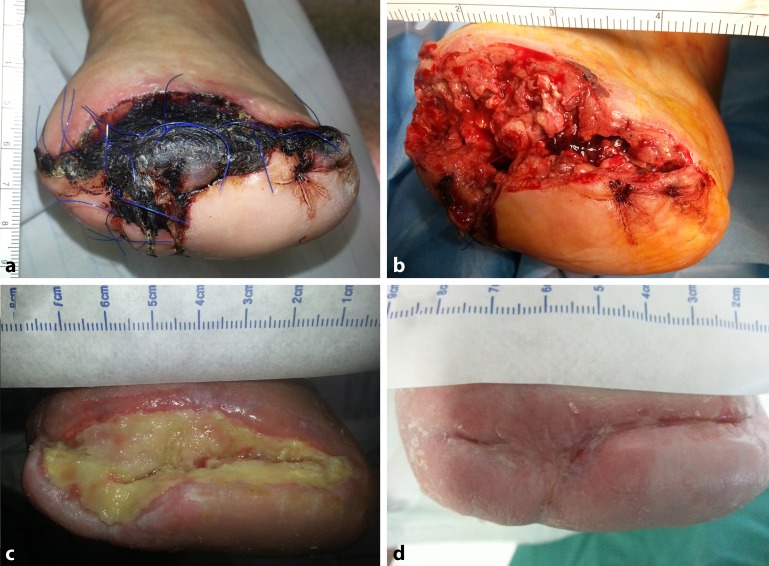
An example of treatment course at the Mainz center. **a** Initial finding involving wound necrosis following forefoot amputation in patient 5. **b** Intraoperative finding following debridement (wound area 29 cm^2^). **c** Interim result after 8 weeks of Omega3 wound matrix therapy. **d** Healed wound following a total treatment duration of 33 weeks (material used: 22 wound matrices à 3 × 7 cm)

Altogether, 10 wounds in 8 patients were treated in the region of the forefoot (*n* = 8, Fig. 2) and the proximal thigh (*n* = 2). The average wound size was 23 ± 18 cm^2^ (range 6–63 cm^2^) and the time to complete wound healing 23 ± 10 weeks (9–41 weeks) on average. To achieve this, a total of 133 Omega3 wound matrices (size 3 × 7 cm) were used (median 17 [7–26] patches/patient).

**Fig. 3:**
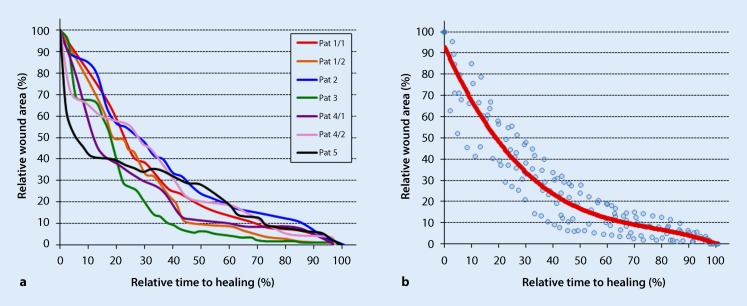
Healing kinetics of the first seven wounds at the Mainz center. **a** Normalized curves of the healing kinetics of seven wounds in five patients, in which the wound size was related to the baseline value and the time related to the total time to healing. **b** A polynomial trend curve was adapted to the individual data matrix in **a** (*Pat *patient). This clearly shows that on average a 50% reduction in wound area could be seen as early on as after 20% of the treatment duration

Based on an evaluation of the photodocumentation, it was possible to also graphically show the kinetics of wound healing for each of the first seven wounds in this study. To improve interpretability, individual wound sizes (baseline surface area = 100%) and the respective time to healing (time of complete healing = 100%) were normalized and presented in the form of a wound-specific healing curve (Fig. 3a). From the resulting dot matrix, a common polynomial trendline was then determined for all curves in a second step (formula: y = −0.0001x^3^ + 0.034x^2^ − 2.8576x + 92.879, Fig. 3b). This non-linear trendline clearly shows that a 50% reduction in wound area could be seen as early on as after 20% of the treatment duration.

Intraoperative swab taking showed colonization with one type of bacteria in five cases (*Escherichia coli*, extended spectrum beta-lactamase-positive *E. coli, Streptococcus agalactiae, Enterobacter cloacae*, and *Staphylococcus aureus*) and multiple colonization in three cases (*S. agalactiae*/*Klebsiella oxytoca*/*Staph. aureus* and *Pseudomonas aeruginosa*/*Enterobacter cloacae, E. cloacae*/*E. coli*/*Enterococcus* species). All patients received targeted antibiotic therapy, with three patients having completed this by the time of discharge, while oral antibiotic therapy was continued in two patients until complete wound healing. An additional effect was observed for the Omega3 wound matrix in that a reduction in pain levels and analgesic use was seen over the course of treatment in all patients on analgesic therapy (wound pain despite DFS). In patient 1 in particular, the (initially high) dose of opioids could be discontinued after 1 week of treatment.

## Experience report from Hamburg-Eppendorf University Hospital

### Methods

**Fig. 4:**
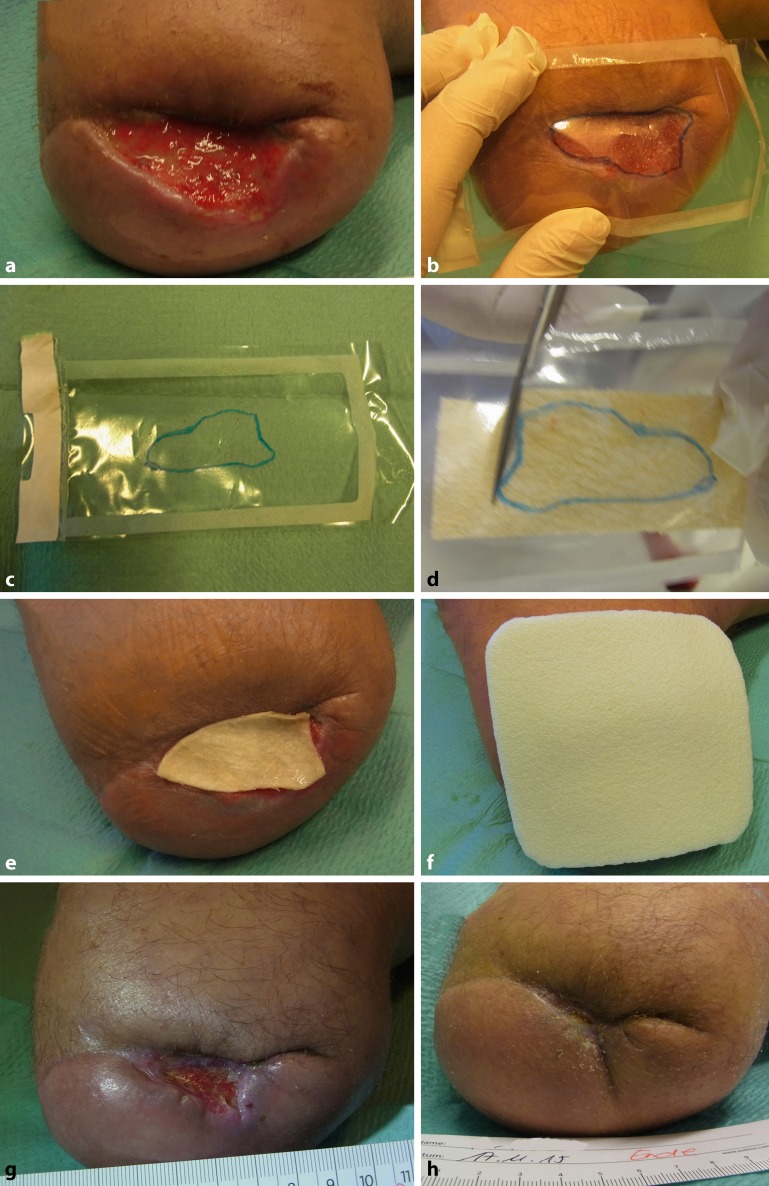
Placement of the omega-3-wound matrix. The size of the wound (**a**) is measured with a plastic sheet (**b**, **c**) and the matrix is shaped accordingly (**d**). After hydration, the matrix is placed on the wound and covered with a polyurethane foam (**f**). During treatment, the wound is progressively remodeling (**g**) until final healing (reepithelialization) is achieved (**h**)

The acellular dermal matrix derived from fish skin (Omega3 wound matrix, Kerecis) was used to treat wounds in 10 patients (10 vascular wounds) between 2015 and 2017 (Table 1). Of the patients eight had peripheral arterial occlusive disease (PAOD) Fontaine stage IV and concomitant DFS, one female patient had extensive vasculitis (MPO [myeloperoxidase], p‑ANCA, cryo-positive) of small, medium, and large vessels in the setting of undifferentiated collagenosis. Ultimately, emergency below-the-knee amputation of both legs was necessary due to advanced gangrene infections. This patient suffered from a persistent wound on the lower leg stump. Another female patient had combined PAOD and small-cell vasculitis in addition to metastatic cancer. Of the patients six had previously undergone endovascular recanalization, while two patients had undergone a pedal bypass. Early occlusion occurred in one patient following bypass surgery, whereby limb preservation was nevertheless possible. Of the patients one had undergone forefoot amputation following revascularization, while ray resections were performed in two cases. A mediasclerotic ABI > 1 was detected in five patients prior to treatment initiation with the Omega3 wound matrix, and the ABI following pedal bypass was 0.87 in one patient and 0.57 in another patient. The ABI in the patient with early graft occlusion was >0.4. At 2–26 months following revascularization, wound healing was absent in all patients despite modern wound treatment with hydroactive wound materials. Wound size varied between 3 cm^2^ and 57.7 cm^2^. No infections were present at the time of (and during) treatment and no patients received antibiotics at the time of treatment. Following thorough wound debridement, the fish-skin matrix that had been cut to the right size (3 × 3.5 cm or 3 × 7 cm, depending on wound size) and soaked in 0.9% saline solution was adapted to the wound size (Fig. 4). The wound was then covered with a polyurethane foam dressing (Biatain Silicone, Coloplast, Humblebæk, Denmark). This dressing was changed at intervals of 2–3 days while leaving the fish-skin matrix in place. Patients underwent treatment every week up to a maximum of nine fish-skin applications. The female patient with early graft occlusion was an exception and to date, she has undergone 17 applications over 12 months after initial treatment success was followed by *Pseudomonas* colonization and subsequent wound enlargement and it became necessary to discontinue Omega3 wound matrix therapy. Following local eradication, treatment with the Omega3 wound matrix was resumed and is currently ongoing. All wounds were photodocumented and measured two-dimensionally. The statistical analysis of patient data and the quantification of wound surface area were carried out using MS EXCEL (Microsoft Corp.) and Fiji/Image J open source software (imagej.nih.gov/ij/), respectively.

### Results

**Fig. 5:**
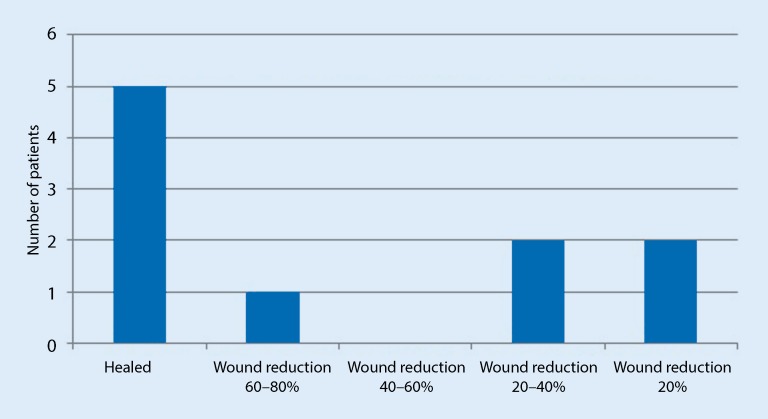
Healing rates/wound reduction in the 10 patients at the Hamburg center. The figure shows the treatment rates of patients in Hamburg, taking treatment days into account

The matrix was completely absorbed within 7 days. It was possible to achieve wound healing in 50% of patients (*n* = 5) within 4 months (60–160 days). The remaining wounds exhibited wound reduction rates of 19–79% within the first 3 months (21–84 days) following treatment initiation (Fig. 5). The largest wound (57.7 cm^2^, present for 25 months prior to treatment initiation) showed a wound reduction rate of 19% within 49 days. In the patient with early pedal bypass occlusion and intermediate wound enlargement following *Pseudomonas* colonization, a wound reduction rate of 67.9% (maximum wound size 46.8 cm^2^, current wound size 15.05 cm^2^) has been achieved to date. No infections or immune reactions occurred during the treatment period. *Pseudomonas* colonization was treated by drying out the wound with a superabsorber (Sorbion Sachet, BSN, Hamburg, Germany) and discontinuing Omega3 wound matrix therapy (Table 1).

## Experience report from Karlsruhe Hospital

### Methods

The use of the Omega3 wound matrix at the Karlsruhe Hospital is reserved for complicated chronic wounds with a certain level of non-reactivity. Efficient exudate and infection control is the first prerequisite of successful application. The fish-skin matrix is preferably applied under sterile conditions in the operating theater following debridement and covered with a polyurethane foam or negative-pressure wound therapy (NPWT); this may need to be repeated several times at weekly intervals. As part of the assimilation process, harmless discoloration and shrinkage of the matrix can be seen after a few days; however, contact with the wound results in improved fibroblast activity and early granulation and if conditioning is successful within the right time window, a mesh graft can be helpful at the end of the process to achieve definitive wound closure.

### Results

**Fig. 6:**
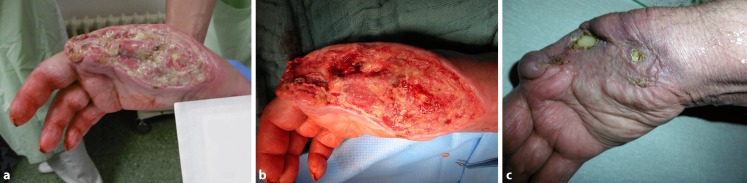
Emergency necrectomy with ray resection of D1 and D2 in the case of intensive catecholamine therapy for sepsis (**a,** **b**). Following Omega3 wound matrix therapy, successful conditioning and mesh graft with virtually complete skin coverage over the course of treatment with the exception of a protruding bone edge in the middle hand (**c**). Hand function can be improved by means of plastic surgery if necessary

**Fig. 7:**
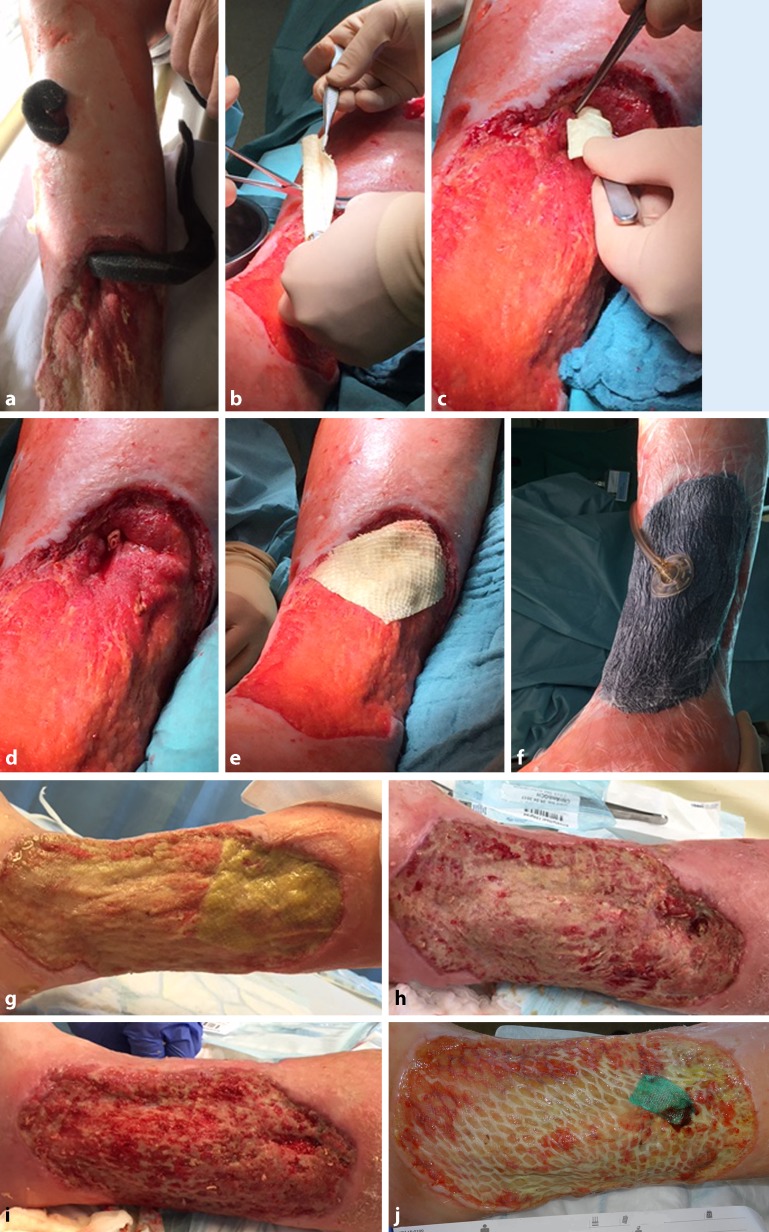
Chronic mixed ulcer involving abscess formation and subsequent persistent long intramuscular soft tissue channel, which was primarily treated with negative pressure wound therapy (NPWT) (**a**). The matrix is introduced as a roll into the channel, while another matrix is placed to cover the surface (**b–e**). Multiple NPWT (**f**); following removal, the wound was protected using a silicone gauze. Successful wound conditioning for a subsequent mesh graft (**g–i**) with healing of the mesh graft (**j**) followed by compression therapy

The matrix has been used highly selectively in only five patients as yet (Table 1) where three of the wounds were on lower limbs and two wounds were on the hand: o hand was treated following borderline amputation due to catecholamine therapy and concomitant radial artery occlusion. The patient, who was multimorbid (liver cirrhosis, chronic myelofibrosis, thromboangiitis, among other disorders), had undergone multiple urological surgical procedures due to perineal Fournier’s gangrene and required intensive care for a prolonged period of time (sepsis, hemofiltration). It was possible to achieve almost complete healing of the wound on the hand (Fig. 6). Another case of use on the hand involved necrosis formation in (protracted) pronounced steal syndrome despite banding of the shunt in dialysis-dependent patients. Of the lower limb wounds two involved persistent, infected lower leg venous ulcers, one of which was an infected mixed ulcer (initially 20 × 15 × 1 cm; W × L × D) accompanied by formation of a deep soft tissue pocket and extensive abscess channel formation in the muscles of a 66-year-old patient with severe postthrombotic syndrome following two instances of deep vein thrombosis and concomitant PAOD. The matrix achieved granulation and healing of the channel (Fig. 7), while the Omega3 wound matrix, an autologous P1 vein bypass, multiple debridement with fasciectomy, and consistent compression therapy were able to condition the ulcer for a mesh graft and achieve its healing. Unfortunately, ulcer recurrence due to insufficient compression and restenosis in the vein bypass were seen at 1‑year follow-up.

Another female patient (aged 101 years) was revascularized by means of percutaneous transluminal angioplasty (PTA) of the superficial femoral artery (SFA) following incision (carried out elsewhere) of a plantar ulcer with progressive, infected (*Staph. aureus*) necrosis in the setting of PAOD and diabetes with compensated renal insufficiency (creatinine 1.9 mg/dl); this patient also underwent two amputations due to progressive necrosis. The matrix was able to stabilize the poorly healing dehiscent wound following closed forefoot amputation, meaning that this elderly patient could be transferred to a rehabilitation center with a preserved limb and infection-free open wound.

In this way, it was possible to achieve complete healing in three patients and stable wound conditions in two patients, one of which (following steal syndrome of the hand) is still under treatment due to poor patient compliance.

Digital photodocumentation was carried out for the reported patients over the course of the inpatient stay; following transfer to an outpatient wound network, photodocumentation was performed using the AKESTES © system (Akestes, Berlin, Germany; [17]). This system enables clinical data collection and area determination by means of pixel marking of the wound area. Thus, the authors’ early experience supports the use of the matrix not only in hard to heal ulcers, but also in persistent cavities on the lower leg and upper limbs.

## Discussion

Impaired wound healing can occur following lower limb amputation in diabetics despite sufficient arterial perfusion, thereby necessitating secondary resection. If impaired wound healing occurs/persists even following secondary resection, the major amputation that then becomes necessary as a result can negate all the preceding efforts at limb preservation (including peripheral bypass). The local situation in the case of wounds in the forefoot area is often further complicated by poor soft tissue conditions and exposed bony segments. Similarly, the same is true for non-diabetic patients with PAOD or vasculitis. Therefore, as discussed in the Introduction, various options to condition the soft tissue or close the wound have been developed, all of which have their individual advantages and disadvantages. Using a marine Omega3 wound matrix, it was possible to achieve wound healing in 25 cases of complicated wounds of the upper and lower extremities in this multicenter experience report. The time required to achieve complete wound healing varies and can take up to 41 weeks in some cases, which demands a certain amount of patience on the part of both the treating physician and the patient; however, patient compliance in the Mainz experience was extraordinarily good, since the patients were happy with the weekly outpatient visits and could be very well guided. In addition to this, the speed of wound healing, particularly in the early treatment phase, was rapid and 50% of the wound area had already healed after the first fifth (20%) of the treatment period. This resulted in a reduction in the time required for wound cleaning and fewer renewals of the Omega3 wound matrix, while this also meant evident treatment success and hence increased satisfaction, particularly among the patients.

Patient compliance was extraordinarily good

A comparison of the wound healing kinetics of patients at the Mainz center with curves from a study on ischemic and non-ischemic wounds also confirmed that the wounds in the Mainz group of patients were adequately perfused/revascularized [18]. By implication, this underscores the need to investigate arterial tissue perfusion and implement appropriate revascularizing measures in the case of local ischemia prior to resection/minor amputation. Another advantage was associated with the use of the Omega3 wound matrix: vacuum conditioning of open wounds is often used at the authors’ centers but only ever in the inpatient setting. By switching to the Omega3 wound matrix, patients could be discharged and the further treatment carried out in the outpatient setting. Furthermore, the side effects associated with prolonged vacuum therapy, such as skin maceration and sealing problems, could be eliminated.

Upper limb wounds could also be treated using this matrix, as was achieved particularly in the Karlsruhe case of infected necrosis of several fingers following catecholamine therapy in pre-existing impaired perfusion. On the other hand, based on early experience, the development of necrosis in the setting of steal syndrome appears to be a less suitable indication.

Over the course of Omega3 wound matrix treatment, the authors also observed that there were other advantageous side effects in some cases in terms of nociception and analgesic use in patients. The most impressive case was of a female patient with a complicated wound in the area of the femoral stump following two secondary amputations, whose initially high opioid use could be completely suspended following 1 week of treatment. Unfortunately, since analgesic use and nociception were not defined endpoints in this study, it is not possible to provide reliable data in this respect; however, the literature reports similar observations of a positive effect for omega-3 fatty acids on nociception, both in animal models [19] and in a case series of five patients [20].

Evidently, there are limitations in the case of *Pseudomonas* colonization, in which case premature absorption of the matrix was observed, while at the same time no positive effect on wound healing was seen at the weekly check-ups. Intermediate wound enlargement was seen in the female patient at the Hamburg wound center and significant wound reduction was seen with the matrix only after eradication of the infection. There are similar (oral) reports from other working groups on the use of Omega3 wound matrix in the case of *Pseudomonas* colonization of the wound.

The limitations of this study lie in the low case numbers despite a multicenter approach and the differing selection criteria and treatment endpoints; however, the primary objective of this investigation was to evaluate the efficacy and usefulness of this preparation in the context of a pilot series. For this reason, no control group was included. Having said that, the initiation of a follow-up study with a control group is currently at the planning stage.

In summary, this initial experience shows the Omega3 wound matrix to be an interesting and effective treatment option for complicated wounds on the lower and upper limbs, an experience confirmed by other centers with similar patient/wound collectives [21]. This is the first clinical multicenter experience report on the use of the Omega3 wound matrix in Germany and the authors were able to successfully treat a total of 25 problem wounds; however, it should be pointed out that while the Omega3 wound matrix clearly represents a specialized treatment option which, according to preliminary data provides additional advantages, such as reduced nociception/analgesic use and the option to perform treatment in the outpatient setting, it requires further clinical evaluation.

## Practical conclusion

- The Omega3 wound matrix is an innovative biological wound dressing derived from cod skin by means of decellularization and a gentle preservation process; it has an extremely high omega-3 fatty acid content and is resistant to bacterial colonization.
- The Omega3 wound matrix is structurally similar to the structure of human skin and permits the ingrowth of fibroblasts and keratinocytes. Omega-3 fatty acids act as a precursor to the synthesis of anti-inflammatory metabolites that have a positive effect on the wound environment.
- The Omega3 wound matrix is suited to the treatment of complicated wounds on the limbs in the context of vascular and diabetes-related diseases as well as chronic venous insufficiency.
- Careful debridement, adequate tissue perfusion, and bacterial control are the essential prerequisites of initial matrix application.
- The advantages of this therapy lie in its outpatient feasibility involving weekly visits and dressing changes, including follow-up application of the wound matrix if required, as well as the local anti-inflammatory and antinociceptive effects of the Omega3 wound matrix.
